# Quantum Hall states observed in thin films of Dirac semimetal Cd_3_As_2_

**DOI:** 10.1038/s41467-017-02423-1

**Published:** 2017-12-22

**Authors:** Masaki Uchida, Yusuke Nakazawa, Shinichi Nishihaya, Kazuto Akiba, Markus Kriener, Yusuke Kozuka, Atsushi Miyake, Yasujiro Taguchi, Masashi Tokunaga, Naoto Nagaosa, Yoshinori Tokura, Masashi Kawasaki

**Affiliations:** 1 Department of Applied Physics and Quantum-Phase Electronics Center (QPEC), the University of Tokyo, Tokyo, 113-8656 Japan; 2 The Institute for Solid State Physics, the University of Tokyo, Kashiwa, 277-8581 Japan; 3 RIKEN Center for Emergent Matter Science (CEMS), Wako, 351-0198 Japan

## Abstract

A well known semiconductor Cd_3_As_2_ has reentered the spotlight due to its unique electronic structure and quantum transport phenomena as a topological Dirac semimetal. For elucidating and controlling its topological quantum state, high-quality Cd_3_As_2_ thin films have been highly desired. Here we report the development of an elaborate growth technique of high-crystallinity and high-mobility Cd_3_As_2_ films with controlled thicknesses and the observation of quantum Hall effect dependent on the film thickness. With decreasing the film thickness to 10 nm, the quantum Hall states exhibit variations such as a change in the spin degeneracy reflecting the Dirac dispersion with a large Fermi velocity. Details of the electronic structure including subband splitting and gap opening are identified from the quantum transport depending on the confinement thickness, suggesting the presence of a two-dimensional topological insulating phase. The demonstration of quantum Hall states in our high-quality Cd_3_As_2_ films paves a road to study quantum transport and device application in topological Dirac semimetal and its derivative phases.

## Abstract (web summary)

Despite many achievements in the topological semimetal Cd_3_As_2_, the high-quality Cd_3_As_2_ films are still rare. Here, Uchida et al. grow high-crystallinity and high-mobility Cd_3_As_2_ thin films and observe quantum Hall states dependent on the confinement thickness.

## Introduction

Topological materials, which are characterized by a non-trivial electronic band topology, have great potential for unprecedented quantum transport phenomena^1–5^. Among them, the topological Dirac semimetal (DSM) has attracted burgeoning attention as emergence of Dirac fermions in three-dimensional (3D) materials^1,2,6–14^. The 3D DSM state is particularly intriguing as a parent phase of exotic topological phases such as 3D topological insulator^3^, Weyl semimetal^4^, and two-dimensional (2D) topological insulator^5,6^, which are realized by symmetry breaking in DSM^1,2,7^. As crystalline materials of 3D DSM, Cd_3_As_2_ and Na_3_Bi have been theoretically suggested^6,8^, and their key electronic structures have been directly confirmed by angle-resolved photoemission and scanning tunneling spectroscopy^7,9–12^. A classification scheme of DSM in terms of the crystal point group symmetry has also been developed^13^.

**Fig. 1:**
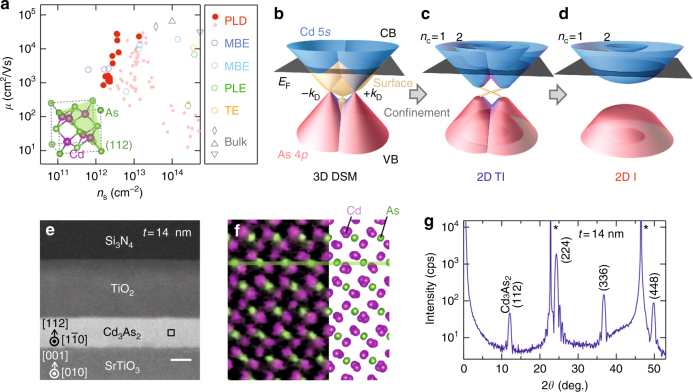
High-crystallinity and high-mobility Cd_3_As_2_ thin films. **a** Experimental trend of electron mobility versus sheet carrier density. Among our films (filled circle) prepared with pulsed laser deposition (PLD), high-quality ones obtained by high-temperature annealing are highlighted with bigger symbols. The mobility reaches a maximum of *μ* = 3 × 10^4^ cm^2^/Vs even at a thickness of *t* = 30 nm, rivaling mobility values for bulk thinned plates (diamond^22^) and nanostructures (triangle^23^ and  inverted triangle^24^), while it intrinsically decreases with reducing to two dimensions. Other films (circle) grown by molecular beam epitaxy (MBE)^18,19^, thermal evaporation (TE)^15^, or pulsed laser evaporation (PLE)^16^ techniques are also plotted for comparison. Inset shows the primary cubic structure of Cd_3_As_2_. **b**–**d** Schematic illustration of the electronic structure evolution from the 3D DSM state. With decreasing the film thickness, subbands are formed due to the quantum confinement, giving rise to two-dimensional topological insulating (2D TI) and trivial insulating (2D I) states depending on the number of inverted subbands^6^. **e** Cross-sectional image of a 14 nm Cd_3_As_2_ film sandwiched between Si_3_N_4_/TiO_2_ cap and SrTiO_3_ substrate. The length of the scale bar is 10 nm. **f** Atomically resolved element map of the boxed region in **e**, shown with the cross-sectional view of the crystal structure. **g** In the x-ray diffraction *θ*–2*θ* scan, Bragg peaks of the (112)-oriented Cd_3_As_2_ film are observed with clear Laue oscillations. The SrTiO_3_ substrate peaks are marked with an asterisk

In this context, fabrication of DSM thin films is of crucial importance for exploring its potential as prototypical topological materials. Unlike other topological materials, however, it has been highly challenging to prepare high-quality DSM films. While Cd_3_As_2_ has been known as a stable II–V type semiconductor from the early period, its film quality has been limited due to the necessity of low-temperature growth for resolving its high volatility (Fig. 1a)^15–19^. Its electronic structure consists of conduction bands (CB) and valence bands (VB) with inverted orbital character, touching to form a 3D Dirac dispersion centered at the Dirac points ±*k*_D_ (Fig. 1b). So far most of the transport studies including surface transport have been reported for bulk samples^20–25^. Tailoring confined Cd_3_As_2_ films with the Dirac dispersion thus opens up new avenues for research of quantized transport in this novel Dirac system such as by gate modulation^26^.

Here we report the development of a growth technique to prepare high-quality Cd_3_As_2_ films and the observation of thickness-dependent quantum Hall effect. The films are epitaxially grown on an oxide substrate with accurately controlled thicknesses, yielding better crystallinity as compared to bulk single-crystals. As theoretically predicted for DSM^6^, successive topological phase transitions to the 2D topological insulator and trivial insulator should occur through the quantum confinement (Fig. 1c, d), if the system becomes 2D in rather thick films. Owing to the large Fermi velocity of the Dirac dispersion, 2D quantum Hall states are actually observed up to such a thick (~23 nm) film.

## Results

### Epitaxial Film Growth

High-quality Cd_3_As_2_ single-crystalline thin films are fabricated by combining pulsed laser deposition and solid phase epitaxy techniques (for details see Methods and Supplementary Figs. 2, 4, and 5). High-temperature annealing made possible by an optimized combination of double capping layers (Si_3_N_4_/TiO_2_) and substrate (SrTiO_3_) significantly improves the crystallinity and the electron mobility of the films. The film triangular lattice is epitaxially grown on the substrate square lattice with aligned in-plane axes, owing to a good match of their projected lattice distances. As confirmed in the transmission electron microscopy image (Fig. 1e, f), the Cd and As atoms are periodically arranged to form the low-temperature Cd_3_As_2_ structure without any discernible crystallographic defects. In the typical x-ray diffraction pattern (Fig. 1g), Bragg peaks of the (112)-oriented Cd_3_As_2_ film are observed with clear Laue oscillations consistent with the designed film thickness. The rocking curve of the film peak has a full width at half maximum of 0.02 degrees, which is sharper than typical values (~0.08 degrees) reported for Cd_3_As_2_ single-crystals^20^.

### Quantum Transport Measurement

**Fig. 2:**
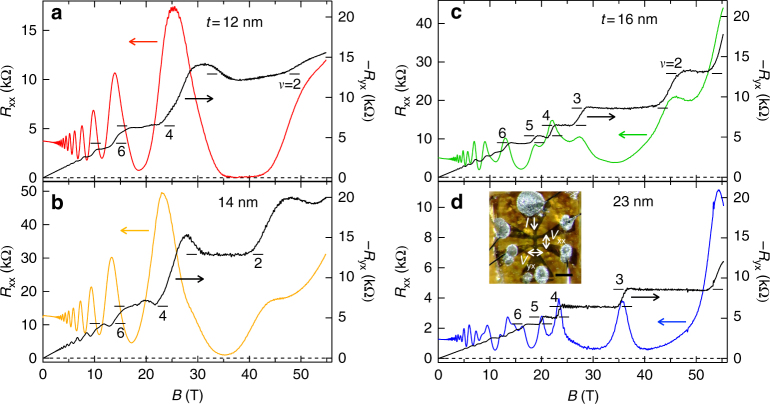
Quantum Hall effect observed in Cd_3_As_2_ films. **a**, **b** High-field magnetotransport for thin films (*t* = 12 and 14 nm) measured at *T* = 1.4 K. The numbers of the horizontal bars represent the filling factor *ν*. The degeneracy factor *s* is determined to be *s* = 2 from the increment of the plateau values. **c**, **d** Same scan for slightly thicker films (*t* = 16 and 23 nm). By contrast, the degeneracy factor is altered to *s* = 1 in these thicker films at high fields. Inset depicts a measured Hall bar with a channel width of 60 μm. The length of the scale bar is 300 μm

Figure 2 summarizes high-field magnetotransport for a set of films with the same carrier density (*n* = 1 × 10^18^ cm^−3^) and different thicknesses (*t* = 12, 14, 16, and 23 nm). Shubnikov-de Haas (SdH) oscillations and corresponding plateau-like structures are resolved from a few teslas in longitudinal resistance *R*_xx_ and Hall resistance *R*_yx_. As the field increases, integer quantum Hall states clearly emerge down to the quantum limit with filling factor *ν* = 2. *R*_xx_ is further suppressed and finally becomes zero. Simultaneously, *R*_yx_ exhibits quantized values over wide field ranges, which are expressed as 1/*R*_yx_ = −*ν*(*e* ^2^/*h*) = −*sn*(*e* ^2^/*h*), with the degeneracy factor *s* and a non-negative integer *n*. A swell around the quantized values confirmed for thinner films is probably an artifact typically appearing in pulsed field measurements of such high resistance samples^27^. Although such deformations of transport data taken in pulsed fields were corrected by calculating the effective current through the sample as detailed in Methods and Supplementary Fig. 6, they cannot be completely removed so far. Absence of the half-integer plateaus suggests that a gap starts to open under the quantum confinement. Furthermore, the degeneracy factor *s* shows a dramatic change depending on the film thickness, governing the appearance of the quantum Hall effect. It is altered from *s* = 2 to 1 when the thickness increases only by 2 nm from *t* = 14 to 16 nm. For clarifying the origin of this change, we analyze the quantum transport in detail.

**Fig. 3:**
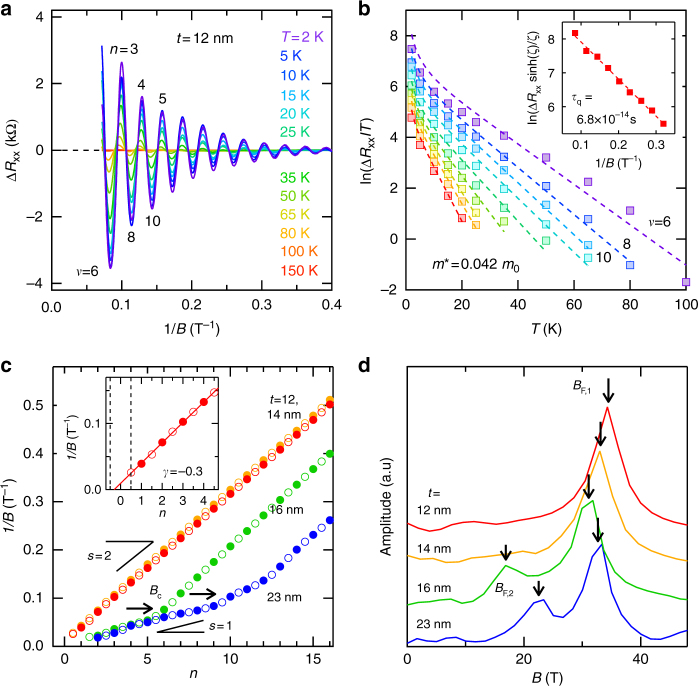
Thickness-dependent quantum transport characteristics. **a** Temperature dependence of the SdH oscillations for the 12 nm Cd_3_As_2_ film, plotted against 1/*B* after subtracting a smooth background from *R*_xx_. **b** Dingle analysis of the oscillation amplitude to obtain effective mass and quantum scattering time (inset). **c** Landau-level fan diagram plotted for the series of films with different thicknesses, and its magnification to evaluate the intercept for the 12 nm film (inset). The integer (half-integer) indices at *R*_xx_ peak (valley) are denoted by closed (open) circles. In the thicker 16 and 23 nm films, spin splitting of the oscillations occurs above the critical field *B*_c_. **d** Fourier transformation of the SdH oscillations below 12 T to extract the oscillation frequencies *B*_F,1_ and *B*_F,2_

The temperature dependence of the SdH oscillations was analyzed for the whole series of Cd_3_As_2_ films, in order to extract effective masses and also quantum scattering times using the Dingle expression^28^. For the 12 nm film, as a typical example, the oscillation amplitude gradually decreases with elevating temperature but remains finite up to about 100 K (Fig. 3a). Its temperature dependence is suitably fitted to the standard Lifshitz-Kosevich formula (Fig. 3b), giving the effective mass of *m* ^*^ = 0.042*m*_0_. This light effective mass originating from the Dirac dispersion is in good agreement with values reported for bulk Cd_3_As_2_^20–25^.

A Landau-level fan diagram is plotted by following maxima and minima in the SdH oscillations (Fig. 3c). The slope dominant in the low-field region corresponds to the primary oscillation from the main Fermi surface as detailed later. In the thicker 16 and 23 nm films, on the other hand, the slope reduces almost by half above the critical field *B*_c_, indicating a change in the degeneracy from *s* = 2 to 1. This change is attributed to spin splitting, not to the lifting of other degeneracies, e.g., of valley or surface states. Quantum confinement is predicted to cause a change in the *g* factor depending on the confinement thickness. In bulk Cd_3_As_2_, spin splitting of the oscillations is observed above *B* ~ 10 T^25^, as in the thick 23 nm film, and the *g* factor is estimated at *g* ~ 15^12,25,29^. Reflecting the existence of other neighboring bands, the *g* factor varies in inverse proportion to quadratic expression of the band gap *E*_g_, according to the Roth equation derived in the second-order *k* ⋅ *p* perturbation theory^30^. The observed thickness dependence of the degeneracy can be thus understood from the rapid opening of the gap due to the confinement. Additionally, the Berry’s phase *ϕ*_B_ can be estimated from the intercept in the fan diagram, based on the expression of the oscillating term^28^. The intercept *γ* is typically about −0.3, which corresponds to a non-trivial Berry’s phase of *ϕ*_B_ ~ 0.4*π*, indicating the presence of relativistic Dirac fermions in the confined dispersion.

From the Fourier transformation of the SdH oscillations, further information about the 2D Fermi surface can be extracted (Fig. 3d). By applying the Onsager relation *A*_F_ = (4*π* ^2^ *e*/*h*)*B*_F_ to the primary oscillation frequency *B*_F,1_, the Fermi surface area *A*_F_ is calculated to be *A*_F_ = 3.3 × 10^−3^ Å^−2^ for the 12 nm film, for example. The dimensional change is also reflected in a clear field-angle dependence of the oscillation and magnetoresistance, as shown in Supplementary Figs. 8 and 10. The Fermi energy *E*_F_, measured from the Dirac points, is estimated to be *E*_F_ = 116 meV by using $k_{\mathrm{F}} = \sqrt {A_{\mathrm{F}}{\mathrm{/}}\pi } = 0.032$ Å^−1^ and *v*_F_ = *ħk*_F_/*m* ^*^ = 8.9 × 10^5^ m/s in the following reported hyperbolic dispersion with an onset energy of *E*_0_ = 50 meV and an energy difference between the conduction band bottom and the Dirac points of *E*_CB_ = 35 meV^6,12^.

1 $$E_{\mathrm{F}} = \hbar v_{\mathrm{F}}\sqrt {k_{\mathrm{F}}^2 + (E_0{\mathrm{/}}\hbar v_{\mathrm{F}})^2} - E_0 - E_{{\mathrm{CB}}}$$

For the thicker 16 and 23 nm films, another peak *B*_F,2_ is detected at lower frequencies, which is ascribed to the subband splitting due to the quantum confinement. The subband electronic structure is also evaluated assuming the same Fermi velocity and onset energy.

## Discussion

**Fig. 4:**
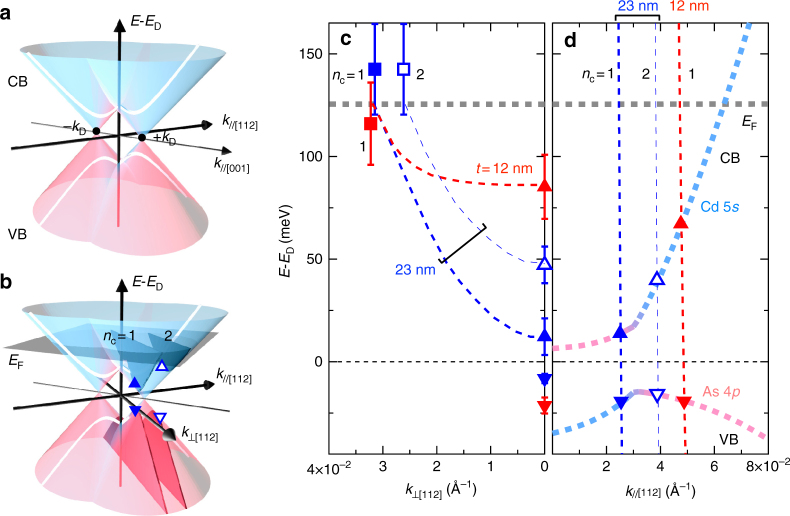
Electronic structures identified from quantum transport. **a** Cross sectional dispersions along the out-of-plane field direction (*k*_||[112]_). **b** Subband formation (*n*_c_ = 1 and 2) within the in-plane direction (*k*_⊥[112]_), for *t* = 23 nm. **c**, **d** Constructed electronic structures measured from the Dirac point energy *E*_D_, are shown for the two typical thicknesses (*t* = 12 and 23 nm) (see Supplementary Fig. 13 for all the thicknesses). In **c**, the obtained Fermi energy *E*_F_ and in-plane Fermi momentum *k*_F_ are plotted for the split subbands (square). The bottom of the conduction band (CB) and the top of the valence band (VB) interpolated from previous calculations^17^ are indicated by an upward and a downward triangle, respectively. The error bars for *E*_F_ were estimated from Eq. (1) by assigning effective mass values obtained using the Lifshitz-Kosevich formula for each filling state *ν* (see Fig. 3b). The structural plot in **d** represents the Bohr–Sommerfeld quantization condition along the film normal direction (Eqs. (2) and (3)), where the crossing point (triangle) of the original calculated band dispersions (dotted lines, left-hand side of Eq. (3))^6^ and the quantization condition curves (vertical dashed lines, right-hand side of Eq. (3)) determines the energy and momentum of the bottom of the split subbands. The orbital character of each subband is also identified, indicating the topological phase transition as represented by the color change of the subband (*n*_c_ = 1) from blue (Cd 5*s*) to pink (As 4*p*). Subband data of *n*_c_ = 1 (2) are distinguished by closed (open) symbols and thick (thin) dashed lines

The various quantum Hall states appearing in the Cd_3_As_2_ films can be comprehensively explained by considering a confinement effect on the original Dirac dispersion as schematised in Fig. 4a, b. For a more quantitative understanding, electronic band structures along the in-plane momentum direction (*k*_⊥[112]_) and the film normal direction (*k*_||[112]_) are summarized in Fig. 4c, d. In Fig. 4c, *k*_F_ and *E*_F_ determined from the above analysis of the SdH oscillations using the dispersion relationship are plotted along the in-plane momentum direction. Here the band edge positions are interpolated from previous calculations^17^. The band gap *E*_g_ is also almost consistent within the error bars with estimations from the *g* factor change (for details see Methods).

The quantum confinement condition along the film normal direction is given by the following formula^31^,

2 $$2k_{||[112]}(E)t + \delta (E) = 2\pi n_{\mathrm{c}}$$

or

3 $$k_{||[112]}(E) = (2\pi n_{\mathrm{c}} - \delta (E)){\mathrm{/}}2t.$$

Here *t* is the film thickness, *n*_c_ is an integer numbering the confined subband, and *δ*(*E*) is the total phase shift at the interfaces (for details see Methods). In the structural plot expressing this relationship in Fig. 4d, the crossing point of the original dispersions (*k*_||[112]_ (*E*)) and the quantization condition curves (*(*2*πn*_c_ − *δ*(*E*))/2*t*) determines energy and momentum of the bottom of the subbands for each thickness. This agrees rather well with the experimental trends including the appearance of the second subband (*n*_c_ = 2) above 16 nm. Reflecting the large Fermi velocity of the Dirac dispersion, the band gap sharply opens when the confinement thickness decreases below 23 nm, giving rise to the dramatic *g* factor change observed in the quantum Hall effect. The band character inversion, which occurs when crossing projected *k*_D_, is also confirmed between *t* = 12 and 23 nm, as denoted by the CB character change from blue (Cd 5*s*) to pink (As 4*p*). In the case of the thick films where the subband is located inside the projected *k*_D_, the gap energy and *g* factor become nearly unchanged.

Novel topological phases derived from the 3D DSM state can be expected for the high-quality Cd_3_As_2_ films. As illustrated in Fig. 1b–d, for example, topological phase transitions to 2D topological (quantum spin Hall) insulator and trivial insulator, as proposed in the original theoretical work of topological states in Cd_3_As_2_^6^, should be induced by the confinement as long as the system remains 2D. Surprisingly, the two dimensionality is maintained up to 23 nm, where the orbital character only of the first subband (*n*_c_ = 1) is inverted, suggesting the presence of a 2D topological insulating phase at this thickness. Below this thickness, another topological phase transition to a trivial insulating phase occurs associated with the sharp change in the *g* factor as confirmed in the thickness-dependent quantum Hall states. Since a magnetic field destroys the 2D topological insulating state by breaking time-reversal symmetry, nonlocal transport and scanning probe microscopy measurements are highly desirable for its further investigation. Applying electric gating, heterostructure fabrication, and chemical doping to such high-quality Cd_3_As_2_ films will open possibilities for further studying quantum transport and device application by tuning Fermi level, hybridization gap, and magnetic interaction in this system.

## Methods

### Epitaxial Film Growth

While Cd_3_As_2_ has been known as a high-mobility semiconductor over half a century^32^, its high-quality thin film growth has been quite challenging. In the 1970s and 80s, rather thicker films than 1 μm were grown by evaporating bulk Cd_3_As_2_ with a heater^15,33–36^ or a high-repetition-rate laser^16,37^, as plotted in Supplementary Fig. 1. More recently, since the topological Dirac semimetal state has been proposed for this system^6^, a more elaborate approach using molecular beam epitaxy (MBE) has realized the epitaxial growth of single-crystalline thin films^17–19,38^. Compared to bulk Cd_3_As_2_, however, their crystallinity and mobility are still limited due to the low-temperature growth. In this situation, we have developed and improved a high-temperature annealing technique with combining pulsed laser deposition (PLD), and obtained high-crystallinity and high-mobility epitaxial thin films comparable to bulk quality. The mobility reaches maximum of *μ* = 3 × 10^4^ cm^2^/Vs for *n* = 1 × 10^18^ cm^−3^, while it intrinsically decreases with reducing dimensions (thickness) from three (≳80 nm) to two (≲40 nm).

Cd_3_As_2_ films and TiO_2_/Si_3_N_4_ capping layers were deposited using KrF excimer laser. Optimization of the capping materials and their combinations has enabled high-temperature annealing of the film, while the idea of adopting a protective layer was already tried for Cd_3_As_2_ growth^15^. A Cd_3_As_2_ polycrystalline target was prepared by mixing 6N5 Cd and 7N5 As shots at a ratio of 3:2, keeping the mixture at 950 °C for 48 h in a vacuum-sealed silica tube, grinding and pelletizing the compound, and then resintering it at 250 °C for 30 h. The three layers were successively deposited on (001) SrTiO_3_ single-crystalline substrates, at room temperature and below a base pressure of 10^−7^ Torr. Typical laser conditions (fluences, repetition rates) were (0.6 J/cm^2^, 10 Hz), (4 J/cm^2^, 20 Hz), and (4 J/cm^2^, 20 Hz), for Cd_3_As_2_, TiO_2_, and Si_3_N_4_, respectively. The shape of the Hall bar, as shown in the inset of Fig. 2d, was defined by employing a stencil metal mask for the successive deposition of the Cd_3_As_2_ and TiO_2_ layers, while the Si_3_N_4_ layer was then deposited on the entire substrate to cover the Hall bar edges. After annealing the sample at 600 °C in air, an (112)-oriented Cd_3_As_2_ film is formed through epitaxial crystallization.

Supplementary Fig. 2 demonstrates the annealing effect probed by x-ray diffraction (XRD). A sample consisting of Cd_3_As_2_ (14 nm) and TiO_2_ (30 nm)/Si_3_N_4_ (200 nm) layers shows only the SrTiO_3_ substrate peaks before annealing. After annealing the sample at 600 °C in air, in stark contrast, (112)-oriented Cd_3_As_2_ film peaks become clearly evident through epitaxial crystallization. Three different periodic components from the respective layers remain observed in both the Kiessig fringes. Many combinations of other substrates (Al_2_O_3_, BaF_2_, CaF_2_, InP, CdTe, mica) and capping materials (Cr:Al_2_O_3_, SiO_2_, MgO, CaF_2_, Si) were also tested, but they resulted in chemical reaction to substrates, cracking of capping layers, or poor crystallinity of the films. As shown in Supplementary Fig. 3, TiO_2_/Si_3_N_4_ capping layer conduction, which is probably due to slightly oxygen-deficient TiO_2_ deposited under the high vacuum, is more dominant at high temperatures above about 100 K, particularly for thinner Cd_3_As_2_ films with higher resistance. At low temperatures, on the other hand, the capping layers become highly insulating and only the Cd_3_As_2_ film conduction remains, ensuring intrinsic quantum transport measurements of the Cd_3_As_2_ films.

Detailed XRD data of the obtained epitaxial film, confirming its high crystallinity and flatness, are summarized in Supplementary Fig. 4. A magnification around the (224) film peak shows clear Laue oscillations consistent with the designed thickness. A rocking curve of the peak has a full width at half maximum (FWHM) of 0.02 degrees, which is sharper than the typical value (0.08 degrees) reported for bulk Cd_3_As_2_ single-crystals^20^. A *ϕ* scan with twelve-fold symmetry reveals that the in-plane [1$\bar 1$0] axis is exactly aligned with the [100] or [010] axes in the substrate, depending on two possible stacking patterns of the six-fold triangular lattice on the substrate square lattice. One reason of the successful epitaxial growth is probably that the projected lattice distance of the Cd_3_As_2_ layer (3.88 Å) has a good match with the SrTiO_3_ one (3.91 Å). The domain size is estimated at about a few tens of microns from the STEM observations on various areas, which are comparable to the channel length scale (~60 μm) but much larger than the magnetic length $l_{\mathrm{B}} = \sqrt {\hbar {\mathrm{/}}eB}$ (~26 nm at 1 T).

Atomic-scale images of the Cd_3_As_2_ single-crystalline film are displayed in Supplementary Fig. 5, taken with cross-section high-angle annular dark-field scanning transmission electron microscopy (HAADF-STEM) and energy dispersive x-ray spectrometry (EDX). Cd and As atoms are periodically arranged without any clear crystallographic defects over a wide area. A shift of Cd atoms present in the low-temperature phase is also detected in the magnified image. From this view direction, it is difficult to determine whether the originally proposed (*I*4_1_ *cd*)^39^ or the recently corrected (*I*4_1_/*acd*)^40^ structure is formed in the film, while both have the similar electronic structure.

### Quantum Transport Measurement

Transport measurements up to 55 T were performed using a nondestructive pulsed magnet with a pulse duration of 37 ms at the International MegaGauss Science Laboratory at the Institute for Solid State Physics of the University of Tokyo. Longitudinal resistance *R*_xx_ and Hall resistance *R*_yx_ were measured on the 60 μm-width multi-terminal Hall bar with flowing a DC current of *I* = 5 μA. In this Hall bar configuration, unexpected effects on the transport such as the current jetting effect in high-mobility semimetals are avoided. Aluminum electrode wires were connected to the Hall bar edges by using an ultrasonic bonding machine and then their connections were reinforced by applying silver paste. Small deformations of transport data taken in the pulsed magnetic fields were corrected based on a simple classic model^27^. In general, when measuring a resistive sample in pulsed fields, a small capacitive component connected parallel to the sample is slightly charged or discharged depending on the resistance change, leading to non-negligible time variation of an effective current through the sample. With the increase in the sample resistance *R*_xx_, the effective current *i*_x_ deviates more from the original set current *I*, and the deformations become more serious. By numerically solving the following differential equation detailed in ref.^27^ with a capacitance of *C* = 4–9 nF, we could calculate the exact current *i*_x_ and obtain data showing negligibly small hysteresis between forward and backward field sweeps, as exemplified in Supplementary Fig. 6.

4 $${\frac{{\mathrm{d}i_{\mathrm{x}}}}{{\mathrm{d}t}} = \frac{{I - i_{\mathrm{x}}(1 + CdR_{{\mathrm{xx}}}{/}\mathrm{d}{\it t})}}{{R_{{\mathrm{xx}}}C}}}$$

Supplementary Fig. 7 demonstrates *R*_xx_ for the 12 nm Cd_3_As_2_ film, measured from 1.4 to 50 K in the pulsed high fields. *R*_xx_ minima at the *ν* = 2 quantum Hall state slowly increase from zero with elevating temperature, which can be well fitted with the standard Arrhenius plot. Obtained high activation energy of Δ = 19 K is ascribed to the unusually high Fermi velocity in Cd_3_As_2_.

At low fields, *R*_xx_ and *R*_yx_ were measured using a Quantum Design Physical Properties Measurement System cryostat equipped with a 9 or 14 T superconducting magnet. Supplementary Fig. 8 plots the data in the low-field region for films of various thicknesses, showing clear Shubnikov-de Haas (SdH) oscillations and Hall plateaus from a few teslas. The degeneracy factor *s* in the quantization formula can be extracted from the increment of the plateau values. Change of the degeneracy from *s* = 2–1 is observed for the 23 nm film at low fields, indicating that spin splitting of oscillations occurs above about 12 T. Apparent degeneracy of *s* = 4 observed for the 16 nm (from *ν* = 16–12) and 23 nm (from *ν* = 20–16) films is ascribable to subband crossing. Corresponding beating pattern due to the existence of another subband can be also confirmed in *R*_xx_ for the 16 and 23 nm films. In contrast to the films below 23 nm, the Hall plateaus in *R*_yx_ become much less pronounced in the 37 nm film and almost completely disappear for the 100 nm film. This suggests that the system gradually changes from two-dimensional (2D) to three-dimensional (3D) around 40 nm.

Supplementary Fig. 9 compares temperature dependence of the SdH oscillations and their analysis to extract effective mass (*m* ^*^) and quantum scattering time (*τ*_q_) following the Dingle expression^28^.

5 $${\frac{{{\mathrm{\Delta }}R_{{\mathrm{xx}}}}}{{R_0}} \propto \frac{{4\zeta }}{{{\mathrm{sinh}}\,\zeta }}e^{ - \pi /\omega _{\mathrm{c}}\tau _{\mathrm{q}}},\zeta = \frac{{2\pi ^2k_{\mathrm{B}}T}}{{\hbar \omega _{\mathrm{c}}}}}$$

Here Δ*R*_xx_/*R*_0_ is the oscillation amplitude normalized by the zero-field resistance and *ω*_c_ = *eB*/*m* ^*^ is the cyclotron frequency. To investigate the main conduction band, the analysis is performed assuming a single band, although the oscillation amplitude at each Landau index is affected by the existence of other subbands for the 16 and 23 nm films. The effective mass is slightly decreased with decrease of the confinement thickness, probably due to the dispersion curvature change associated with the gap opening.

Supplementary Fig. 10 shows angular-dependent SdH oscillations in *R*_xx_, measured also with a conventional superconducting magnet. When the applied magnetic field is tilted from out-of-plane (*θ* = 0°) to in-plane (90°) direction in the 23 nm film, the oscillation period as well as the amplitude is substantially reduced. A gradual dimensional change from a cylindrical (2D) Fermi surface to a spherical (3D) Fermi surface is observed between 23 and 100 nm, consistent with the thickness dependence of Hall plateaus in Supplementary Fig. 8. For the 37 nm film, a cylindrical but corrugated (quasi-2D) Fermi surface is confirmed in the beginning of the dimensional change. Weak corrugation can be confirmed also for the 23 nm, but the 23 nm film rather closer to 3D shows much higher second frequency *B*_F,2_ than the 16 nm one, eliminating the possibility of the neck orbit as a cause of *B*_F,2_. Along with this dimensional change, considerably large negative magnetoresistance probably due to so called chiral anomaly^14^ is also observed for the *B* || *I* configuration (*θ* = 90°) on the Hall bar.

Landau-level fan diagrams magnified around the origins are shown in Supplementary Fig. 11 for all the thicknesses. The Berry’s phase *ϕ*_B_ can be estimated from the intercept *γ* − *δ* in the fan diagram, on the basis of the following expression of the oscillating term in Δ*R*_xx_^28^.

6 $$\frac{{{\mathrm{\Delta }}R_{{\mathrm{xx}}}}}{{R_0}} \propto {\mathrm{cos}}\left[ {2\pi (B_{\mathrm{F}}{\mathrm{/}}B - \gamma + \delta )} \right]$$

Here *B*_F_ is the SdH oscillation frequency, *γ* is the phase factor expressed as *γ* = 1/2 − *ϕ*_B_/2*π*, and *δ* is the phase shift being zero in two dimensions. For the 12 and 14 nm films, the intercept is about −0.3, which corresponds to the non-trivial Berry’s phase of *ϕ*_B_ ~ 0.4*π*. In Cd_3_As_2_, the Berry’s phase extracted from the intercept has been highly scattered and controversial^17,20,21,24,25,41–43^. According to the recent theoretical calculation^43^, when the Fermi energy is located above the saddle point of the two Dirac dispersions as in the cases of the previously reported carrier densities, the non-trivial phases at ±*k*_D_ are canceled out $\left( {\phi _{\mathrm{B}} = \phi _{{\mathrm{B}}, + k_{\mathrm{D}}} + \phi _{{\mathrm{B}}, - k_{\mathrm{D}}} = 0} \right)$. In our two-dimensional case, however, the non-trivial Berry’s phase remains finite without the cancellation within the confined subband, indicating the presence of the relativistic Dirac fermions therein. For the 16 and 23 nm films, modulated behavior due to formation of the other subbands makes it difficult to evaluate the intercept accurately.

Further low-temperature quantum transport was also measured using a dilution refrigerator. In Supplementary Fig. 12, *R*_xx_ and *R*_yx_ taken at 40 mK for the 12 nm film are compared to 2 K ones, showing no major difference between them. So far, no more fine structures such as fractional quantum Hall states are confirmed in the present samples even at this ultra-low temperature up to 14 T.

### Construction of electronic structure

The band gap *E*_g_ is also estimated from the *g* factor change in the following Roth equation, which is derived in the second order of the *k* ⋅ *p* perturbation theory^30^.

7 $$g = 2 - \frac{2}{3}\frac{{E_{\mathrm{p}}{{\Delta }}}}{{E_{\mathrm{g}}(E_{\mathrm{g}} + {{\Delta }})}}$$

Here *Δ* is the spin–orbit splitting energy (0.27 eV)^29^ and *E*_p_ is the energy equivalent of the principal interband momentum matrix element (−0.68 eV). *E*_g_ is estimated at ≳110, ≳110, ~55, and ~30 meV for the 12, 14, 16 and 23 nm films, by using the above parameters and respective *g* factors (≲5, ≲5, ~9, and ~15). These electronic structures parameters are summarized in Table 1.

**Table 1:** Parameters of electronic structures for Cd_3_As_2_ films of various thicknesses.

*t* (nm)	m* (*m*_0_)	*E*_F_ (meV)	*k*_F_ (Å^−1^)	*g*	*E*g (meV)
12	0.042	116	0.032	≲5	≳110
14	0.038	129	0.032	≲5	≳110
16	0.038	114	0.031, 0.023	~9	~55
23	0.035	143	0.032, 0.026	~15	~30
100 (bulk)	0.049	134	0.037	~15	0

The total phase shift at both interfaces was estimated as a function of the binding energy *E*_B_ by assigning band gap values *E*_g_ and chemical potential differences Δ*ϕ* of SrTiO_3_, TiO_2_, and Cd_3_As_2_ to the following empirical formula^44^.

8 $$\begin{array}{*{20}{l}} {\delta (E)} \hfill & \hskip-8pt = \hfill &\hskip-7pt {2\,{\mathrm{arcsin}}\sqrt {\frac{{E_{{\mathrm{g}},{\mathrm{SrTiO}}_3} - {\mathrm{\Delta }}\phi _{{\mathrm{SrTiO}}_3,{\mathrm{Cd}}_3{\mathrm{As}}_2} - E_{\mathrm{B}}}}{{E_{{\mathrm{g}},{\mathrm{SrTiO}}_3}}}} } \hfill \\ {} \hfill & + \hfill & {2\,{\mathrm{arcsin}}\sqrt {\frac{{E_{{\mathrm{g}},{\mathrm{TiO}}_2} - {\mathrm{\Delta }}\phi _{{\mathrm{TiO}}_2,{\mathrm{Cd}}_3{\mathrm{As}}_2} - E_{\mathrm{B}}}}{{E_{{\mathrm{g}},{\mathrm{TiO}}_2}}}} - 2\pi } \hfill \end{array}$$

### Data Availability

The data supporting the plots within the paper and its Supplementary Information File are available from the corresponding author upon reasonable request.

## Electronic Supplementary Material

Supplementary information
