# Supplementary material for: Quantum Hall states observed in thin films of Dirac semimetal Cd3As2
Source: Nat Commun. 2017 Dec 22;8:2274. doi: 10.1038/s41467-017-02423-1 (PMC5741620; doi:10.1038/s41467-017-02423-1)
Supplement: Supplementary file 1 — Supplementary information [file 41467_2017_2423_MOESM1_ESM.pdf]

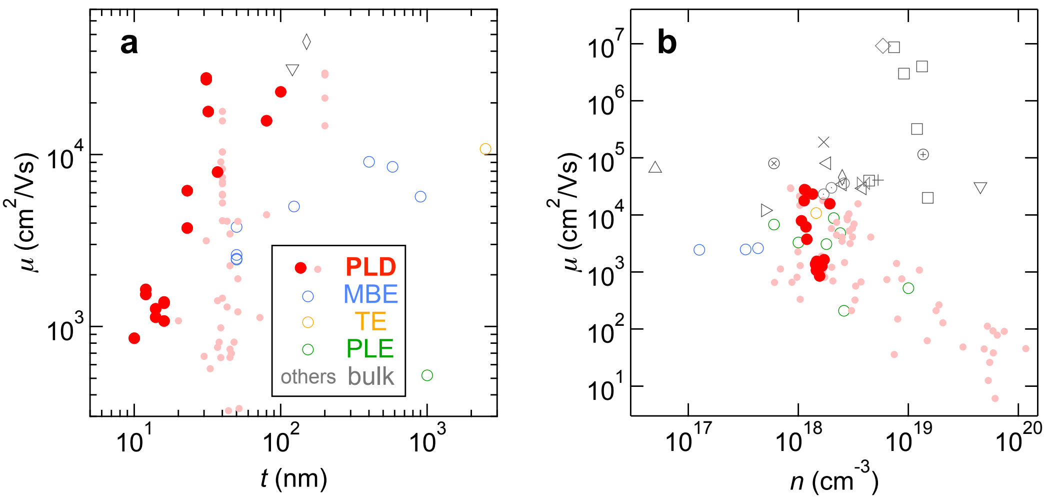

Supplementary Figure 1: **Trends of mobility and carrier density in Cd_3As_2 samples.** Electron mobility versus (**a**) sample thickness and (**b**) volume carrier density summarized for Cd_3As_2 film and bulk samples fabricated to date. Our films prepared with pulsed laser deposition (PLD) are indicated by closed circles, and in particular, high quality ones obtained by high temperature annealing are highlighted with bigger circles. Other films previously grown by molecular beam epitaxy (MBE) [1, 2], thermal evaporation (TE) [3], or pulsed laser evaporation (PLE) [4, 5] techniques are represented by open circles. Bulk samples grown such as by Cd flux or chemical vapor transport are denoted by other gray symbols (◊ [6], △ [7], ▽ [8], □ [9], ◇ [10], ◁ [11], ▷ [12], ⋈ [13], × [14], + [15], ⊙ [16], ⊗ [17], and ⊕ [18]).

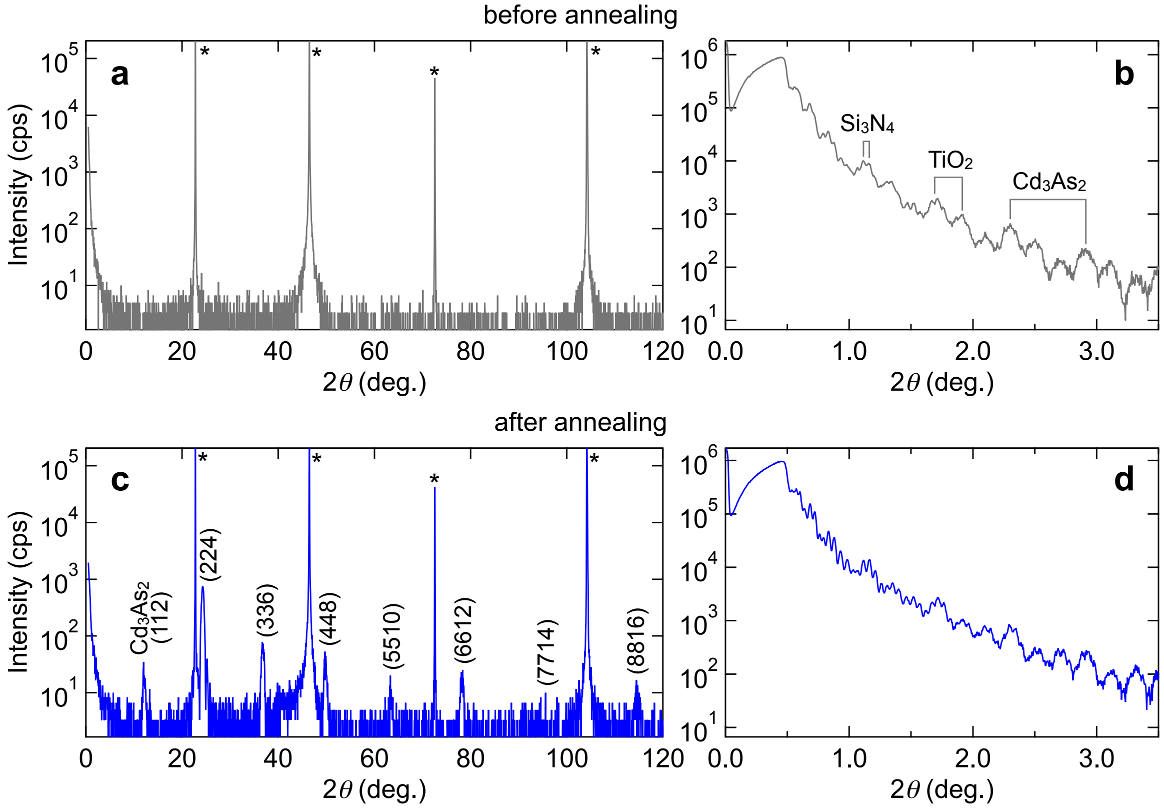

Supplementary Figure 2: **Annealing effect probed by x-ray diffraction.** (**a**) Typical x-ray diffraction θ–2θ scan and (**b**) small angle reflectivity curve of a Cd_3As_2 film capped with TiO_2/Si_3N_4 layers before annealing. The $SrTiO_3$ substrate peaks are marked with an asterisk. (**c**) and (**d**) Results of the same film after high-temperature annealing.

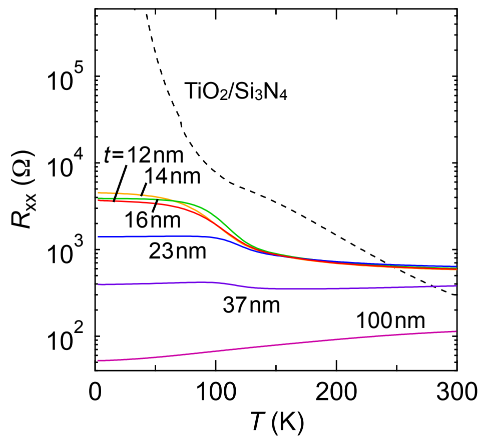

Supplementary Figure 3: **Temperature dependence of the sample resistance.** Temperature dependence of $R_{\rm xx}$ is plotted for the capped Cd_3As_2 films with various thicknesses, also compared to a sample consisting only of the TiO_2 / Si_3N_4 capping layers.

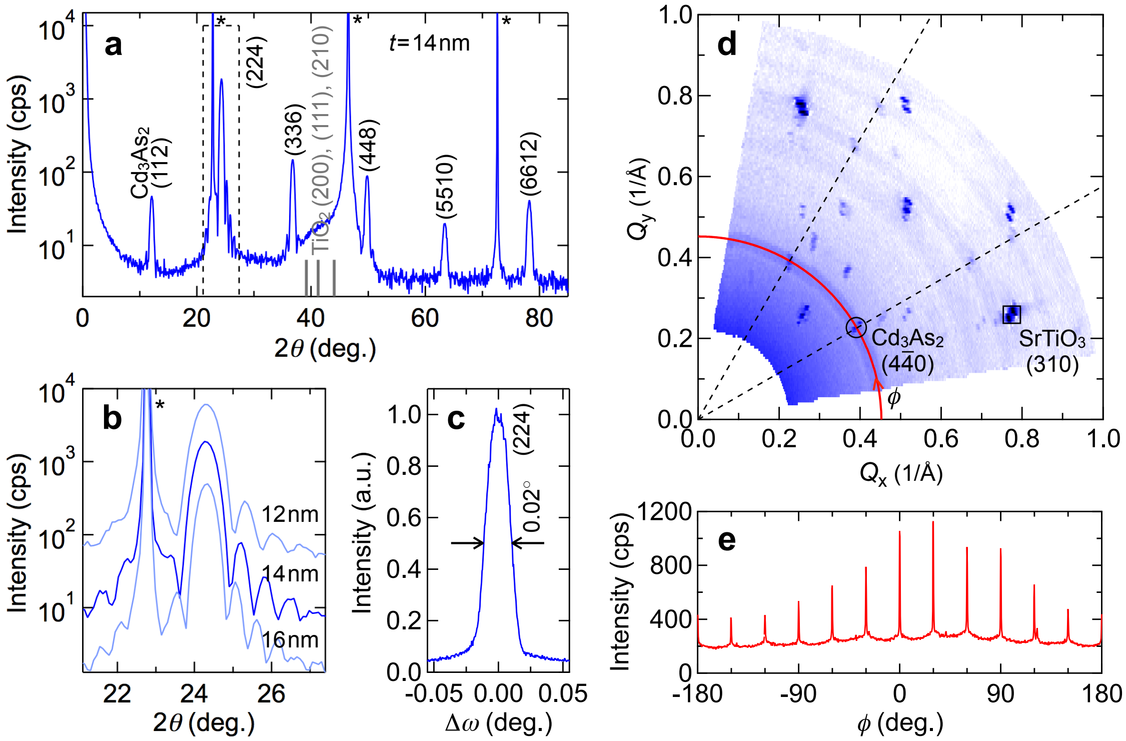

Supplementary Figure 4: **X-ray diffraction characterization of the Cd_3As_2 films.** (**a**) Lower speed θ–2θ scan of the 14 nm Cd_3As_2 film. The substrate peaks are marked with an asterisk. A background hump around 40 degrees is ascribable to polycrystalline TiO_2 peaks. (**b**) Magnification of the (224) peak and its Laue oscillations, compared to those for 12 and 16 nm films. (**c**) Rocking curve of the (224) film peak with a full width at half maximum (FWHM) of 0.02 degrees. (**d**) In-plane reciprocal space mapping and (**e**) ϕ scan of the $(4\bar{4}0)$ peak, showing locked in-plane orientation of the film.

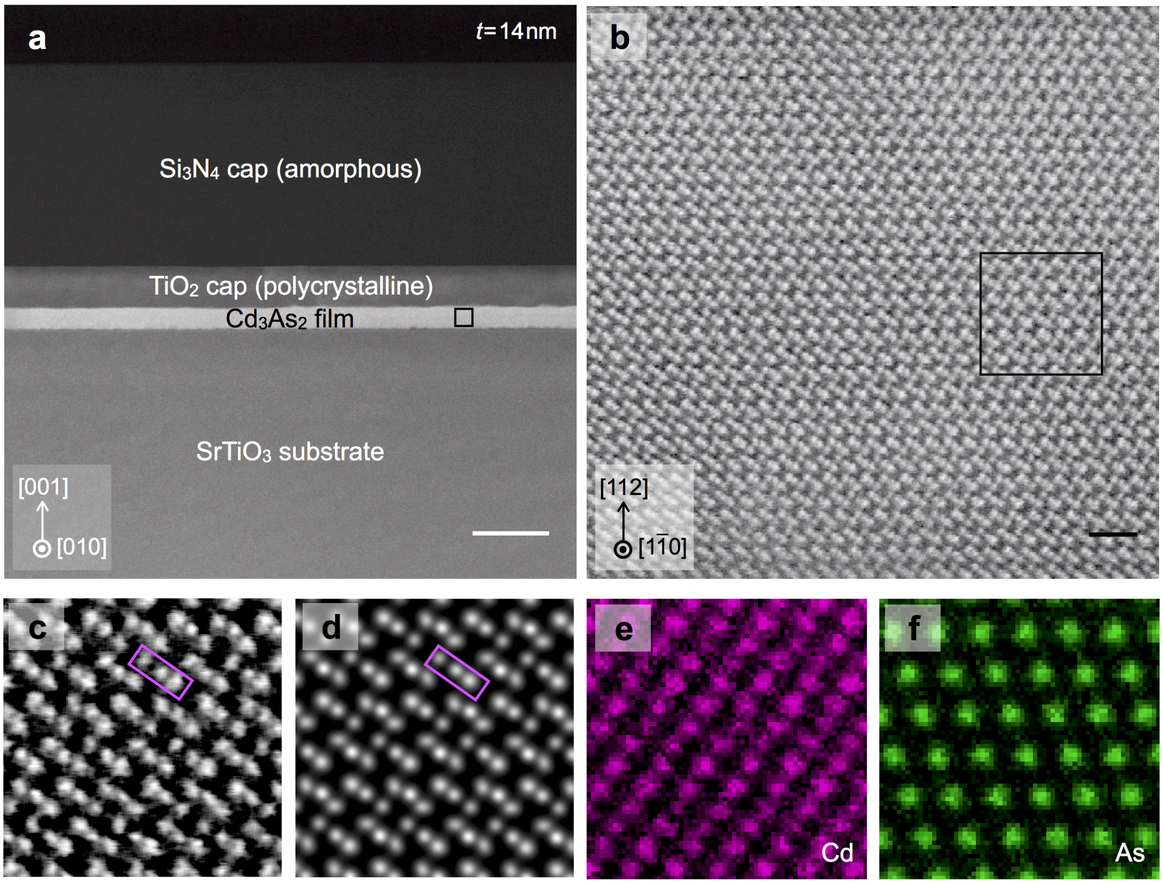

Supplementary Figure 5: **Transmission electron microscopy characterization of the Cd_3As_2 film.** (**a**) Overall picture of the heterostructure and (**b**) magnified view of the Cd_3As_2 film of the boxed area in (**a**), taken with cross-section high-angle annular dark-field scanning transmission electron microscopy (HAADF-STEM). The lengths of the scale bars are 50 nm and 1 nm, respectively. (**c**) Higher-resolution magnified image of the Cd_3As_2 film of the boxed area in (**b**), (**d**) simulated image for the low-temperature structure of Cd_3As_2 ($I4_1/acd$), and corresponding element maps taken with energy dispersive x-ray spectrometry (EDX) for (**e**) Cd L and (**f**) As K edges. Shift of Cd atoms peculiar to the low-temperature structure is clearly confirmed as indicated in the box.

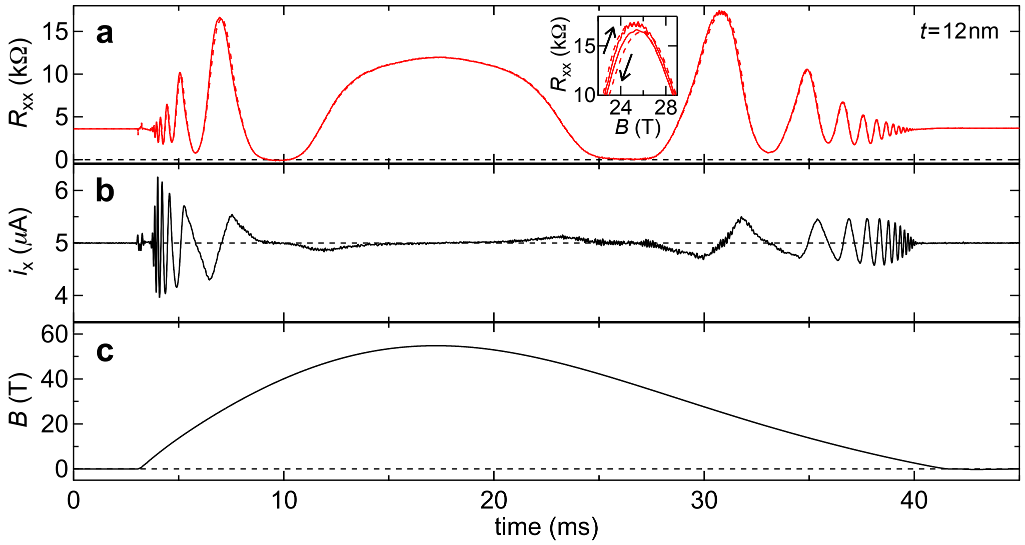

Supplementary Figure 6: **Correction of data taken in the pulsed magnetic fields.** (**a**) Raw (dashed line) and corrected (solid line) R_{xx} curve for the 12 nm film, taken in a single scan of pulsed field shown in (**c**). The inset shows magnified data resolving hysteresis. The correction was made using a classic model [19] as expressed in Eq. S1, in which a small capacitive component of 5 nF is taken into consideration to calculate (**b**) a time-variable actual current i_{x} though the sample.

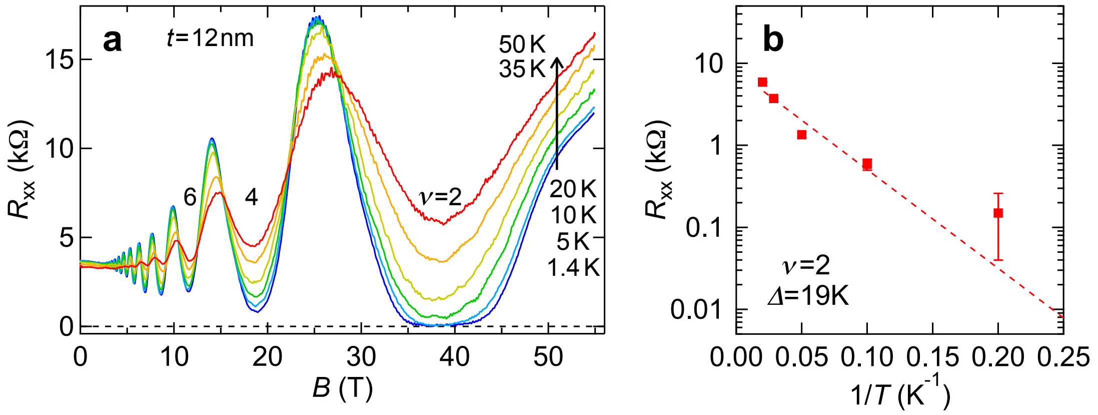

Supplementary Figure 7: **Temperature dependence of quantum Hall effect.** (**a**) Temperature dependence of R_{xx} for the 12 nm Cd_3As_2 film in high magnetic fields. (**b**) Arrhenius plot of the R_{xx} minima for the $\nu = 2$ quantum Hall state. The error bars were estimated from maximum and minimum values in the upward and downward sweeps.

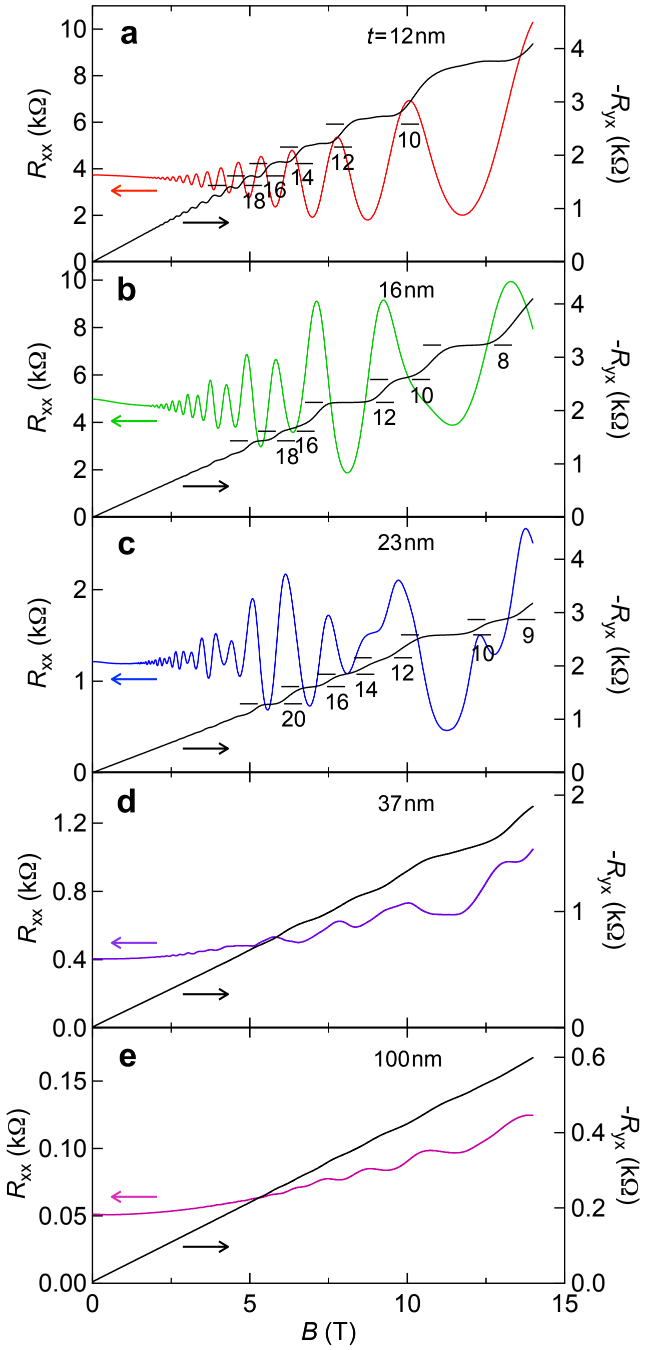

Supplementary Figure 8: **Low-field magnetotransport dependent on the confinement thickness.** $R_{\rm xx}$ and $R_{\rm yx}$, taken using a conventional superconducting magnet at 2 K, are plotted for the (**a**) 12 nm, (**b**) 16 nm, (**c**) 23 nm, (**d**) 37 nm, and (**e**) 100 nm films. Shubnikov-de Haas (SdH) oscillations are resolved for a field as low as a few teslas, and corresponding clear Hall plateaus are also confirmed for the films below 23 nm. The numbers with the horizontal bars represent -1/$R_{\rm yx}$ values in the unit of e^2/h.

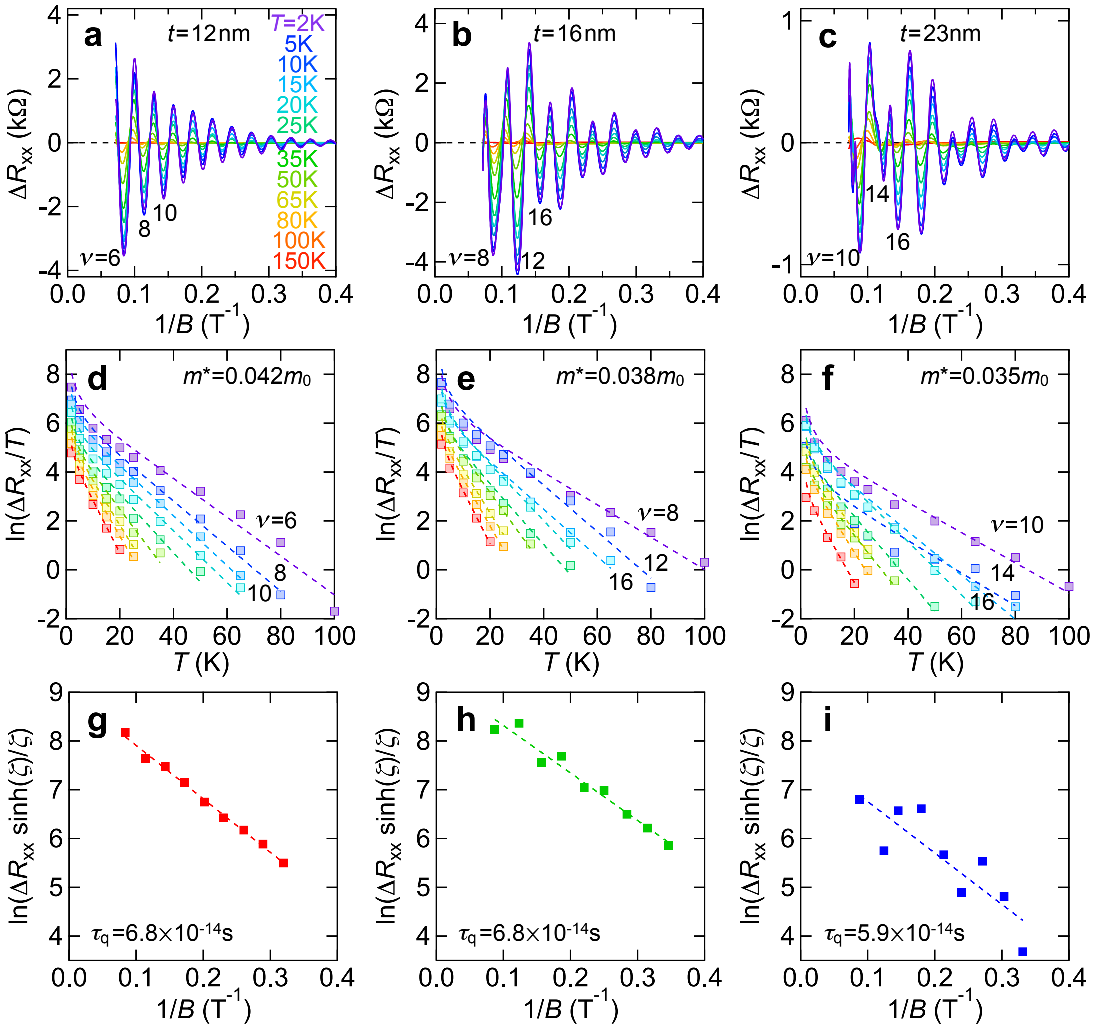

Supplementary Figure 9: **Temperature dependence of the SdH oscillations and their analysis.** (**a**)-(**c**) Temperature dependence of the SdH oscillations, plotted as a function of $1/B$ after subtracting a smooth background. (**d**)-(**f**) Lifshitz-Kosevich analysis of the oscillation amplitude for each filling state. The broken curves are fits to the formula, yielding an effective mass of $0.035 \sim 0.042 m_0$. (**g**)-(**i**) Dingle plots of the oscillation amplitude. A quantum scattering time of $\tau_q = 6 \sim 7 \times 10^{-14}$ s is extracted from the slope of the linear fit, corresponding to Dingle temperature of $T_D = 18 \sim 21$ K.

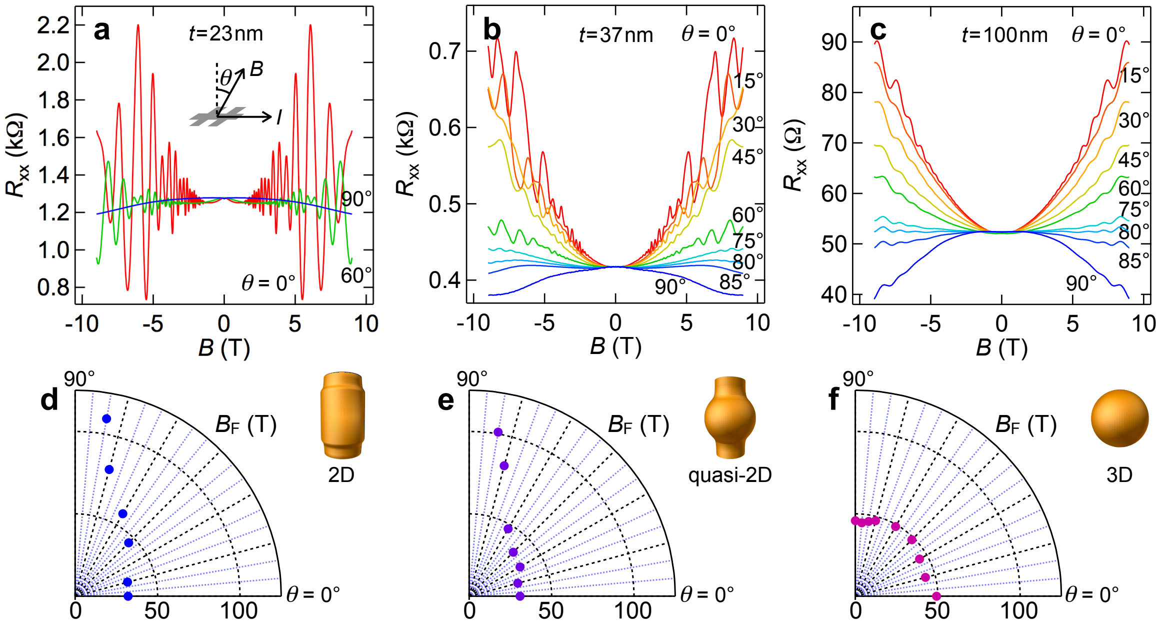

Supplementary Figure 10: **Field angle dependence of the SdH oscillations reflecting the dimensional change.** Evolution of the SdH oscillations measured with changing the field direction from $\theta = 0^\circ$ ($B \perp I$) to 90° ($B \parallel I$) for the (**a**) 23 nm, (**b**) 37 nm, and (**c**) 100 nm films at 2 K. (**d**)-(**f**) Polar plots showing the angle dependence of the oscillation frequency for the three films. As contrasted to the two-dimensional (2D) behavior in the 23 nm film, quasi-2D behavior appears at the 37 nm film and it changes into three-dimensional (3D) at the 100 nm film.

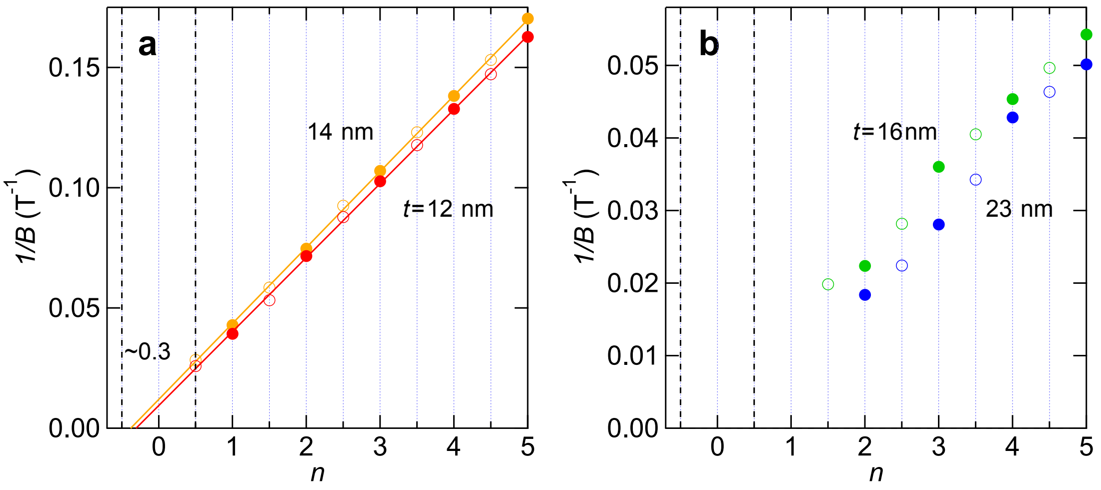

Supplementary Figure 11: **Landau-level fan diagrams magnified around the origins.** Landau-level fan diagrams plotted for (**a**) the thinner (t = 12, 14 nm) and (**b**) thicker (t = 16, 23 nm) films. The integer (half-integer) indices at R_{xx} peak (valley) are denoted by a closed (open) circles. For the 16 nm and 23 nm films, spin splitting of the oscillations already occurs in this field range.

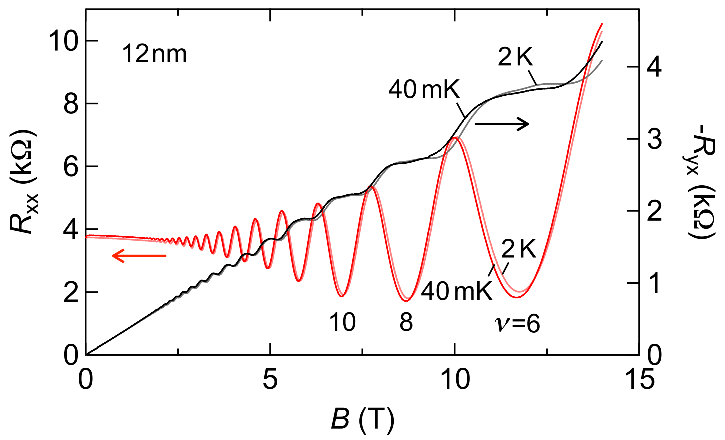

Supplementary Figure 12: **Ultra-low temperature magnetotransport measured at 40 mK.** R_{xx} and R_{yx}, taken using a dilution refrigerator with the base temperature, are plotted for the 12 nm Cd_3As_2 film. It does not show any significant difference from that at 2 K.

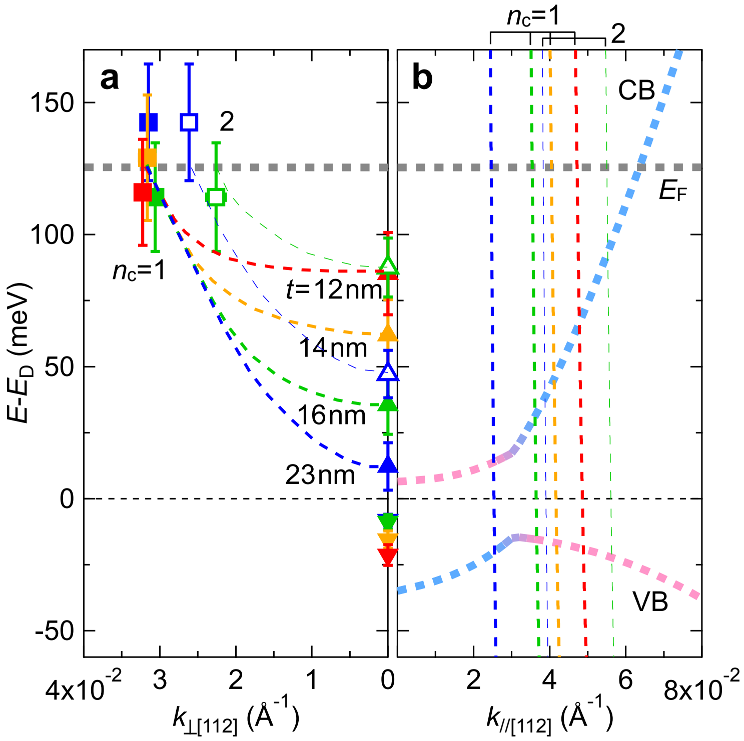

Supplementary Figure 13: **Electronic structures under the quantum confinement.** Constructed electronic structures are plotted for all the thicknesses ($t = 12, 14, 16,$ and 23 nm), while in Fig. 4 in the main text they are shown only for the two typical thicknesses ($t = 12$ and 23 nm) for clarity. In (**a**), the Femi energy and in-plane momentum ($k_{\perp[112]}$) obtained from the quantum transport analysis are denoted by a square. The bottom of the conduction band (CB) and the top of the valence band (VB) are indicated by an upward and a downward triangle. Subband data ($n_c = 1$ and 2) are distinguished by closed and open symbols, respectively. (**b**) Quantum confinement condition along the film normal direction ($k_{\parallel[112]}$).

Supplementary References

[1] Cheng, P. *et al. New J. Phys.* **18**, 083003 (2016).

[2] Zhao, B. *et al. Sci. Rep.* **6**, 22377 (2016).

[3] Żdanowicz, W., Żdanowicz, L., Portal, J. C. & Askenazy, S. *Thin Solid Films* **61**, 41–50 (1979).

[4] Dubowski J. J. & Williams, D. F. *Appl. Phys. Lett.* **44**, 339 (1984).

[5] Dubowski J. J. & Williams, D. F. *Can. J. Phys.* **63**, 815–818 (1985).

[6] Moll, P. J. W. *et al. Nature* **535**, 266–270 (2016).

[7] Feng, J. *et al. Phys. Rev. B* **92**, 081306(R) (2015).

[8] Zhang, E. *et al. ACS Nano* **9**, 8843–8850 (2015).

[9] Liang, T. *et al. Nat. Mater* **14**, 280–284 (2015).

[10] Zhao, Y. *et al. Phys. Rev. X* **5**, 031037 (2015).

[11] Narayanan, A. *et al. Phys. Rev. Lett.* **114**, 117201 (2015).

[12] Zhang, C. *et al. Nat. Commun.* **8**, 13741 (2017).

[13] Rosenberg, A. J. & Harman, T. C. *J. Appl. Phys.* **30**, 1621–1622 (1959).

[14] Cao, J. *et al. Nat. Commun.* **6**, 7779 (2015).

[15] He, L. P. *et al. Phys. Rev. Lett.* **113**, 246402 (2014).

[16] Rogers, L. M., Jenkins, R. M. & Crocker, A. J. *J. of Phys. D: Appl. Phys.* **4**, 793 (1971).

[17] Weber, C. P. *et al*. *Appl. Phys. Lett.* **106**, 231904 (2015).

[18] Chen, Z.-G. *et al*. *Nano Lett.* **15**, 5830–5834 (2015).

[19] van der Burgt, M. *et al*. *Physica B* **177**, 409 (1992).
